# Identification of *PKD2* mutations in human preimplantation embryos *in vitro* using a combination of targeted next-generation sequencing and targeted haplotyping

**DOI:** 10.1038/srep25488

**Published:** 2016-05-06

**Authors:** Song-Chang Chen, Xiao-Li Xu, Jun-Yu Zhang, Guo-Lian Ding, Li Jin, Bei Liu, Dong-Mei Sun, Chang-Lin Mei, Xiao-Nan Yang, He-Feng Huang, Chen-Ming Xu

**Affiliations:** 1 Institute of Embryo-Fetal Original Adult Disease Affiliated to Shanghai Jiao Tong University School of Medicine, 910 Hengshan Road, Shanghai 200030, P.R. China; 2 International Peace Maternity and Child Health Hospital, Shanghai Jiao Tong University School of Medicine, 910 Hengshan Road, Shanghai 200030, P.R. China; 3 BGI-Shenzhen, Shenzhen, 518083, P.R. China; 4 Hangzhou Genomics Institute, 478 Wensan Road, Hangzhou 310012, P.R. China; 5 Key Laboratory of Reproductive Genetics (Zhejiang University), Ministry of Education, 1 Xueshi Road, Hangzhou 310006, P.R. China; 6 Division of Nephrology, Kidney Institute of CPLA, Changzheng Hospital, Second Military Medical University, Shanghai,200003, P.R.China

## Abstract

Here, we evaluate the applicability of a new method that combines targeted next-generation sequencing (NGS) with targeted haplotyping in identifying *PKD2* gene mutations in human preimplantation embryos *in vitro*. To achieve this goal, a proband family with a heterozygous deletion of c.595_595 + 14delGGTAAGAGCGCGCGA in exon 1 of the *PKD2* gene was studied. A total of 10 samples were analyzed, including 7 embryos. An array-based gene chip was designed to capture all of the exons of 21 disease-related genes, including *PKD2*. We performed Sanger sequencing combined with targeted haplotyping to evaluate the feasibility of this new method. A total of 7.09 G of data were obtained from 10 samples by NGS. In addition, 24,142 informative single-nucleotide polymorphisms (SNPs) were identified. Haplotyping analysis of several informative SNPs of *PKD2* that we selected revealed that embryos 3, 5, and 6 did not inherit the mutation haplotypes of the *PKD2* gene, a finding that was 100% accurate and was consistent with Sanger sequencing. Our results demonstrate that targeted NGS combined with targeted haplotyping can be used to identify *PKD2* gene mutations in human preimplantation embryos *in vitro* with high sensitivity, fidelity, throughput and speed.

Preimplantation genetic diagnosis (PGD) is a specialized technique that can reduce the risk of having a child with a particular genetic or chromosomal disorder. PGD is used to detect genetic changes in embryos that are created by using assisted reproductive techniques such as *in vitro* fertilization. To perform PGD, a small number of cells are taken from the embryos and are tested for certain genetic changes. Only normal embryos are implanted in the uterus to initiate a pregnancy [http://ghr.nlm.nih.gov/handbook/testing/uses]. Since the world’s first birth of healthy baby girls by PGD in 1990^1^, there have been thousands of families affected by heritable genetic disorders who have used this technology to give birth to normal babies. At present, more than 150 types of single-gene disorders can be detected by PGD.

The *PKD2* (MIM:173910) gene is located on the long (q) arm of chromosome 4 at position 22.1. The official name of this gene is “polycystic kidney disease 2 (autosomal dominant)”. More than 75 mutations in the human *PKD2* gene have been identified in patients with polycystic kidney disease. These mutations are responsible for approximately 15% of all cases of autosomal dominant polycystic kidney disease (ADPKD), the most common type of PKD (http://ghr.nlm.nih.gov/gene/PKD2). ADPKD, which is generally a late-onset multisystem disorder characterized by bilateral renal cysts, affects 1 in 500 to 1,000 people. Cyst growth leads to enlarged kidneys, which results in kidney failure. The signs and symptoms of ADPKD typically begin in adulthood, and approximately 50% of individuals with ADPKD have end-stage renal disease (ESRD) by the age of 60 years. Except for hemodialysis and kidney transplantation, which are used in treating ESRD, there is no effective therapy to delay PKD progression (http://www.ncbi.nlm.nih.gov/books/NBK1246/). Therefore, the selection of healthy embryos for uterine transfer by PGD has become the most effective way to avoid ADPKD transmission.

Current and emerging technologies for PGD include single-cell PCR, fluorescence *in situ hybridization* (FISH), array comparative genomic hybridization (aCGH), single-nucleotide polymorphism (SNP) arrays and next-generation sequencing (NGS). The disadvantages of single-cell PCR and FISH are laborious, expensive and time consuming and usually require a specific locus and pedigree. The aCGH, SNP arrays and NGS are the latest single-cell genomic methodologies with high sensitivity, throughput and speed and can replace PCR and FISH^2^.

Because only one or a few cells from each human preimplantation embryo can be used for embryo biopsy, problems such as contamination, allele drop-out (ADO), amplification failure and preferential amplification (PA) are inevitable in single-cell PCR^3^. Comparatively speaking, whole-genome amplification (WGA) combined with single-cell genomics methodologies have become a more effective way to reduce the incidence of these types of problems^4^,^5^,^6^. Another elegant approach called karyomapping uses genome-wide SNP arrays, and the SNP haplotypes are analyzed using SNP genetic markers^7^. Karyomapping is theoretically capable of mapping haplotypes for all of the genes in any family^8^, as the SNPs are distributed widely in the genome with high density, and the analysis can be automated^9^. However, genome-wide karyomapping is still expensive for identifying the inheritance of single-gene defects.

In recent years, NGS technology has developed rapidly and has established various applications in the field of medical testing that can be applied to aneuploidy screening, DNA copy-number aberrations, carriership of balanced chromosome rearrangements, single-gene disorders, *de novo* segmental copy-number aberrations, *de novo* base mutations and mitochondrial mutations. NGS has been conducted with high accuracy, good stability, high throughput and lower cost^10^,^11^,^12^,^13^.

Therefore, we considered using NGS to detect mutations with the WGA products of a single cell from human preimplantation. An array-based gene chip was designed to capture all of the exons of several genes of interest associated with heritable genetic disorders. This gene chip also allowed us to obtain information on approximately 2,000 SNP (minor allele frequency [MAF] > 0.3) loci regions around each gene and the X and Y chromosome-specific regions. To avoid of inevitable ADOs, we subsequently combined targeted NGS with targeted haplotyping to identify *PKD2* gene mutations in preimplantation embryos from a proband family *in vitro*. To evaluate this technology, we used the Sanger DNA sequencing method to confirm the results obtained from targeted NGS and targeted haplotyping.

## Results

### An overview of the performance of targeted NGS

We obtained 7.09 G of raw data from 10 samples. On average, 93.3% of the data were high-quality data (>Q20 and N ≦ 5), and approximately 78.8% of the data yielded clean reads that could be uniquely matched to the NCBI human genome reference build 37. The average utilization rate of the data was 76.6%. In addition, the average coverage and depth of the targeted region were 92% and 108.7-fold, respectively. On average, we detected 24,142 SNPs (Table 1).

### Targeted NGS and haplotype analysis results

**Figure 1:**
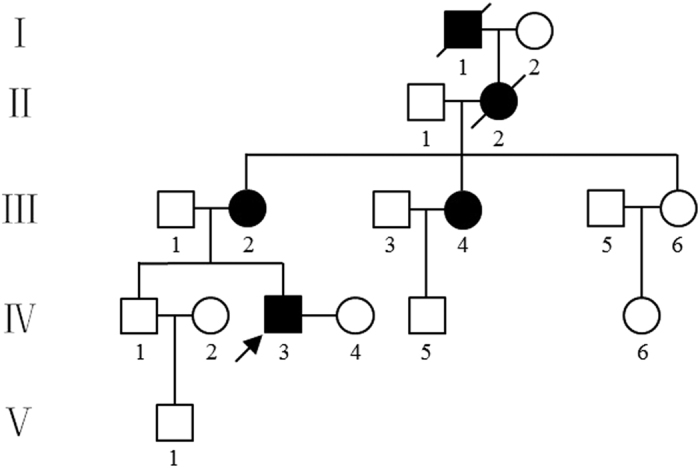
The pedigree of a family affected by autosomal dominant polycystic kidney disease. IV_3_: proband.

For our analysis, we selected SNP loci in which the husband was heterozygous and both the mother and wife were homozygous. The pedigree of the family was shown in figure 1. There were approximately 123 informative SNPs distributed in the upstream, intragenic and downstream regions of the *PKD2* gene. Among these sequences, 24 were informative SNPs, whereby the SNP genotype of father was heterozygous, and both the paternal grandmother and mother were homozygous but had different SNP genotypes. The remaining 99 informative SNPs had the same SNP genotypes. The results of the targeted NGS and the haplotype analysis of the pedigree are shown in Tables 2 and 3. Table 2 shows that embryos 1, 2, 4, and 7 are abnormal due to the inheritance of haplotype 1, a mutated haplotype, from the father. In addition, as shown in Table 3, embryos 3, 5, and 6 were normal because they inherited haplotype 2, a normal haplotype, from the father.

### Sanger DNA sequencing and haplotype analysis results

Seven informative SNP candidates were selected from the SNPs in targeted NGS (4 upstream, 2 intragenic and 1 downstream) to perform Sanger DNA sequencing. However, only 5 informative SNPs (2 upstream, 2 intragenic and 1 downstream) were stable and were of high quality for haplotype analysis in embryos. The results from Sanger DNA sequencing of the 5 SNP informative loci are shown in Table 4 and in the Supplementary information (Fig. S1).

**Figure 2:**
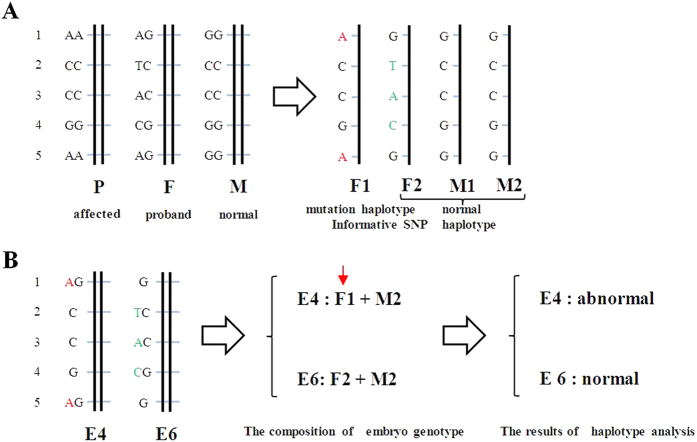
Principles of family-specific haplotyping and embryo analysis based on SNP genotyping of Sanger sequencing. Family-specific haplotype analysis based on SNP genotyping of the parents and paternal grandmother in five candidate SNPs. “P” represents “paternal grandmother”, “F” represents “father”, “M” represents “mother”, “F1” represents “haplotype 1 of the father”, “F2” represents “haplotype 2 of the father”, “M1” represents “haplotype 1 of the mother” and “M2” represents “haplotype 2 of the mother”. The five candidate SNPs were selected from the SNPs of the targeted NGS that were also confirmed to have stable and quality results in the direct detection of embryos by Sanger sequencing. The five SNP genotypes of the father were heterozygous, and both the paternal grandmother and mother were homozygous but had different SNP genotypes. By Mendelian analysis of the SNP genotypes of the parents and the paternal grandmother, it is possible to identify the four parental haplotypes. The SNP loci colored in red and green represent the informative SNPs that could determine the unique haplotype. The informative SNPs colored in red were from haplotype 1 of the father, and the green SNPs were from haplotype 2 of the father (**A**). “E4” represents “embryo 4”, and “E6” represents “embryo 6”. The two embryos illustrate the principle of embryo haplotype analysis. The results from the embryo SNP genotypes were obtained from Sanger sequencing. The SNP loci coding embryos 1 and 5 are represented by two letters because they were heterozygous. The SNP loci coding embryos 2, 3, and 4 are represented by one letter, likely due to ADO. Embryo 4 was determined to have an abnormality that resulted from inheritance of mutation haplotype 1 from the father. Embryo 6 was normal (**B**).

As illustrated in Table 4 and Fig. 2, embryos 1, 2, 4, and 7 indeed carried one or two mutation SNP haplotypes that were inherited from the father, and embryos 3, 5, and 6 were normal and carried 1 to 3 informative SNP loci in haplotype 2 of the normal paternal haplotypes.

### Direct sequencing results of the *PKD2* gene mutation

The results of direct mutation detection of the *PKD2* gene c.595_595 + 14delGGTAAGAGCGCGCGA are shown in Table 5 and in the Supplementary information (Fig. S2). It was impossible to complete direct mutation detection by targeted NGS because the mutation was located in exon 1 with poor capture efficiency. Moreover, the results from the direct mutation detection by Sanger sequencing were mismatched with the haplotypes of embryos 2 and 7, likely due to the ADO. Therefore, it is obvious that the application of NGS for high-quality SNPs in *PKD2* and in combination with haplotype analysis is more effective for PGD.

## Discussion

In this study, we used targeted NGS to perform tests on 10 samples from one family and analyzed the data with targeted haplotyping using the informative SNPs as genetic markers. The haplotypes of the *PKD2* gene in 7 embryos were consistent with the results of the traditional Sanger sequencing method. We demonstrated that targeted NGS combined with targeted haplotyping could be used in the identification of *PKD2* gene mutations in human preimplantation embryos *in vitro*. Because our capture custom array contained thousands of SNPs with a wide distribution across the genome, it was very suitable for haplotype analysis without having to screen for genetic markers in different people. The analysis is also automated. In addition, we designed X and Y chromosome-specific regions in our chip for the sex identification of some X-linked genetic diseases. In doing so, we accidentally found that embryo 6 was missing an X chromosome, a finding that was consistent with the results of aCGH. This finding suggested that we should focus on the X and Y chromosome-specific regions to avoid sex chromosomal aneuploidy abnormalities in embryo screening. In addition, the method we used in our research is also suitable for the detection of a large number of samples from multiple different families with diseases in a single experiment. These samples can be simultaneously sequenced as long as each sample is ligated to a different library adapter. Each gene chip can capture more than 10 samples, and each sample only requires approximately 1 GB of sequencing data. However, this new method requires approximately one week to complete sequencing and data analysis, which cannot be used for fresh embryo transfer.

In addition to *PKD2*, targeted NGS combined with targeted haplotyping can be used in other single-gene disorders for PGD, such as *DMD* or *PKD1* (data shown in supplementary Tables S2–S4), of which the normal embryos were also selected with 100% accuracy. However, targeted NGS has poorer capture efficiency in exon 1 and in some gene regions with high GC content. Coincidentally, in this study, the mutation position of the *PKD2* gene was located in exon 1; therefore, direct mutation detection was impossible. The same phenomenon also occurred in direct mutation detection using the Sanger DNA sequencing method because of ADO (see Supplementary information (Table S5). If the mutation occurred beyond these special regions, direct mutation detection by targeted NGS can normally be used to assist with the analysis and to further reduce the risk of detection failure. The results from targeted NGS demonstrated that ADO was random but was not acting on each heterozygous informative SNP locus. Hence, heterozygous informative SNP loci were more suitable for haplotype analysis in embryos, and the homozygous informative SNP loci could be used as a reference (at least to identify one of the haplotypes). Moreover, on the basis of the special custom gene chip, we can further design one or multiple genes located on each chromosome for haplotype analysis, which detects most of the chromosomal copy-number aberrations that result from mis-segregation during meiosis I. This method would be a great significant for embryo screening.

## Conclusions

Targeted NGS combined with targeted haplotyping can be used to identify *PKD2* gene mutations in human preimplantation embryos *in vitro*.

## Methods

### Study materials

A total of 10 samples from a pedigree were collected at the Women’s Hospital School of Medicine Zhejiang University (Fig. 1). Samples from the husband (proband), the proband’s mother, the proband’s wife (normal) and 7 embryos were collected. The proband was diagnosed with renal cysts via B ultrasound four years ago in one examination. His mother has been affected by PKD for approximately 20 years. Mutation detection of the *PKD1* and *PKD2* genes using the Sanger method demonstrated that both the proband and his mother carried a heterozygous deletion of c.595_595 + 14delGGTAAGAGCGCGCGA in exon 1 of *PKD2*, but the mutation was not present in the proband’s wife. This study was performed with the approval of the Ethics Committee of the International Peace Maternity and Child Health Hospital of Shanghai Jiao Tong University School of Medicine. The study was conducted in adherence with the Declaration of Helsinki. Written informed consent was obtained from the proband and his family.

### Genomic DNA extraction and WGA

In this study, we collected 4 ml of peripheral blood with EDTA anticoagulant from every family member and used a QIAamp DNA Blood Mini Kit (Qiagen, Hilden, Germany) to extract the genomic DNA. The cell from the embryo used for PGD was biopsied from a 6- to 8-cell human cleavage-stage embryo on day three after IVF. Then, we used laser-assisted microdissection of the zona pellucida for embryo biopsy and performed WGA with a Qiagen WGA kit (150345#REPLI-g Single Cell Kit (96), QIAGEN/A) as soon as possible.

### Capture array design, library construction and targeted NGS

A custom capture array (Nimblegen SeqCap EZ Choice XL Library, Roche, Swiss) was used for the PGD of single-gene disorders. The total capture region was 3.5 M, including 21 common genes associated with genetic disorders. The capture region of this gene chip was divided into three parts. The first part was the coding region, including the alternative splice region and other gene regions associated with genetic diseases. This grouping was applied to direct mutation detection. The second part was a region with an approximately 2,000 SNP (MAF > 0.3) locus within a 2 M range on each side of the targeted gene. The SNPs (MAF > 0.3) were derived from the international 1000 Genomes Project (International HapMap Project) database^14^. Because of the large number of polymorphisms, it was feasible to use these SNPs as generic genetic markers for individual identification. The third part was the X and Y chromosome-specific region for sex identification.

Fragmentation of the DNA into 200- to 300-bp pieces was completed using an ultrasonoscope (Covaris S2, Massachusetts, USA). Next, 1 μg of purified DNA (quantified via NanoDrop) was treated with T4 DNA polymerase, T4 phosphonucleotide kinase and the Klenow fragment of the *Escherichia coli* DNA polymerase to fill 5′ overhangs and remove 3′ overhangs. Terminal A residues were added following a brief incubation with dATP and the Klenow 3′–5′ exo-enzyme using standard Illumina protocols^15^. Adapter oligonucleotides from Illumina (single reads) were ligated to the ends. After the ligation was completed, successful adapter ligation was confirmed in a four-cycle PCR using a high-fidelity polymerase with PCR primers containing a custom-synthesized barcode sequence (8 bp) as the sample index signature. PCR was used to generate a library for further analysis, and the DNA adapter-ligated and indexed fragments from the 10 libraries were pooled and hybridized to the capture array for 72 h. After hybridization and washing, the DNA fragments bound to the array were eluted using 300 ml of elution buffer (Qiagen, Valencia, CA, USA) for each array. A gasket (Agilent) was applied and placed on an in-house-constructed thermal elution device for 20 min at 95 °C. We repeated this process once, adding 200 ml of elution buffer (Qiagen). After hybridization of the sequencing primer, base incorporation was performed on Illumina HiSeq2500 Analyzers (following the manufacturer’s standard cluster generation and sequencing protocols) for 90 cycles of sequencing per read to generate paired-end reads including 90 bp at each end and 8 bp of the index tag. Image analysis and base calling were performed using the Illumina Pipeline.

### Data filtering, analysis and SNP identification

After sequencing, the primary data were obtained using the Illumina Pipeline, and the indexed primers were used to identify the different reads from different samples in the primary data. Only the reads that were perfectly matched to the theoretical adapter-indexed sequences and reads that matched the theoretical primer-indexed sequences with a maximum of three mismatches were considered to be acceptable reads. We then removed a few unqualified sequences from the primary data using a local dynamic programming algorithm, which included low-quality reads, which were defined as reads that contained more than 10 percent Ns in the read length, 50% reads with a quality value of less than 5 and with an average quality of less than 10 and adapter sequences that included the indexed sequence. The remaining sequences were considered to be clean reads for further analysis.

The clean reads with a length of 90 bp were then compared to the reference human genome from the NCBI database (Build 37) using the BWA (Burrows Wheeler Aligner) Multi-Vision software package^15^. SNPs and indels were identified using the SOAPsnp software^29^ and the GATK Indel Genotyper (http://www.broadinstitute.org/gsa/wiki/index.php/, The Genome Analysis Toolkit), respectively. Previously identified SNPs were determined using the NCBI dbSNP or HapMap databases. Known disease-causing mutations were identified using the Human Gene Mutation Database at the Institute of Medical Genetics in Cardiff (HGMD, http://www.ghmd.cf.ac.uk/) or from mutations previously reported in the literature.

### Principles of haplotype analysis and sex identification of embryos

**Figure 3:**
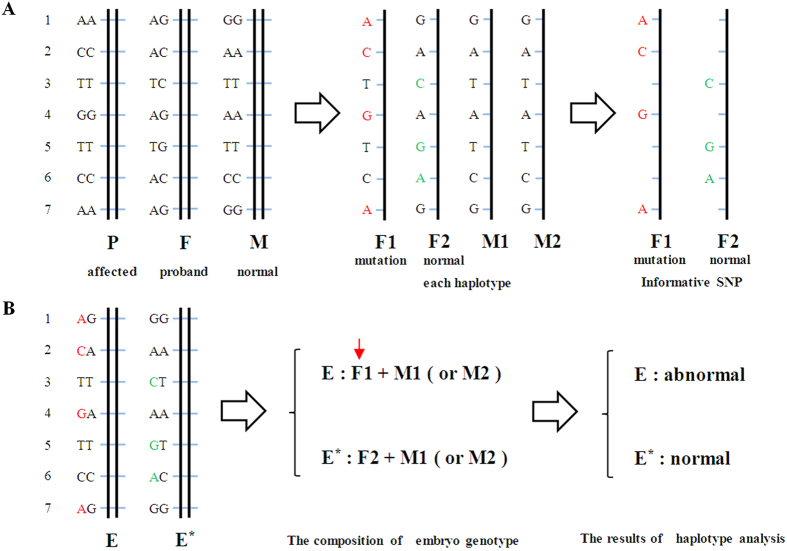
Principles of haplotype analysis. A family-specific haplotype analysis based on SNP genotyping of the parents and the paternal grandmother. “P” represents “paternal grandmother”, “F” represents “father”, “M” represents “mother”, “F1” represents “haplotype 1 of the father”, “F2” represents “haplotype 2 of the father”, “M1” represents “haplotype 1 of the mother” and “M2” represents “haplotype 2 of the mother”. The seven closely linked SNPs were selected from the NGS SNP loci and were coded 1 to 7. The SNP genotype of the father was heterozygous, and both the paternal grandmother and the mother were homozygous. The proband and his mother were both affected by PKD, while the proband’s wife was normal. By Mendelian analysis of the SNP genotypes of the parents and the paternal grandmother, it is possible to identify the four parental haplotypes. Furthermore, it is easy to determine F1 as a mutation haplotype and a disease-causing haplotype and F2 as a normal haplotype. The SNP loci colored in red represent the informative SNPs in F1 that can determine the unique haplotype of F1. The SNP loci colored in green represent the informative SNPs in F2 that can determine the unique haplotype of F2. By contrast, the SNP loci colored in black are defined as invalid SNPs (**A**). “E” represents “embryo”. According to the SNP genotypes of E and E*, we can infer that E inherited haplotype 1 of the father because it carried the informative SNPs of F1. Similarly, we can infer that E* inherited haplotype 2 of the father because it carried the informative SNPs of F2. Therefore, we can define E as abnormal and E* as normal (**B**).

SNPs from the paternal and maternal haplotypes were analyzed to identify informative SNP loci. Informative SNP loci were those loci in which the SNPs of the two parents were in the same location and that one parent was homozygous and the other was heterozygous. This method was used to identify unique haplotypes. The informative SNPs were then assembled to form a unique haplotype according to the SNP information from the child’s samples based on Mendelian analysis. According to the linked informative SNPs of the embryo, we could identify the paternal or maternal origin of each haplotype of the embryo and map the inheritance of these haplotypes. In addition, we could determine whether the embryo inherited the mutation haplotype (Fig. 3). Furthermore, we could identify the sex of the embryo based on the depth and coverage of sequencing from X and Y chromosome-specific regions, See Supplementary information (Table S1).

## Additional Information

**How to cite this article**: Chen, S.-C. *et al*. Identification of *PKD2* mutations in human preimplantation embryos *in vitro* using a combination of targeted next-generation sequencing and targeted haplotyping. *Sci. Rep*. **6**, 25488; doi: 10.1038/srep25488 (2016).

## Supplementary Material

Supplementary Information

## Figures and Tables

**Table 1:** Overview of the performance of targeted NGS.

Sample	Raw data (MB)	Data utilization (%)	Coverage of target region (%)	Mean depth of target region (×)	Number of SNP loci (the depth of sequencing ≥10×)
P	491.57	85.6	96.29	55.37	26793
F	437.37	87.2	96.60	50.70	25628
M	486.26	86.1	96.38	57.90	27336
E1	976.01	80.7	92.98	163.99	22535
E2	810.43	79.8	94.35	139.89	21981
E3	695.76	81.0	92.90	131.72	24320
E4	914.73	80.1	91.92	181.01	23799
E5	652.88	78.9	90.78	80.44	22984
E6	908.28	79.1	72.84	142.64	23117
E7	716.39	79.9	95.07	83.81	22930

P represents “Paternal grandmother”, F represents “Father”, M represents “Mother”, E represents “Embryo”.

**Table 2:** Targeted NGS and haplotype analysis results of the pedigree.

Embryo	F	F
	haplotype 1	haplotype 2
	upstream/intragenic /downstream	
E1	11/0/13	0/0/0
E2	11/0/13	0/0/0
E3	0/0/0	0/0/0
E4	10/0/6	0/0/0
E5	0/0/0	0/0/0
E6	0/0/0	0/0/0
E7	9/0/12	0/0/0

The SNP genotype of the father is heterozygous, and both the paternal grandmother and mother are homozygous but have different SNP genotypes.

**Table 3:** Targeted NGS and haplotype analysis results of the pedigree.

Embryo	F	F
	haplotype 1	haplotype 2
	upstream/intragenic /downstream	
E1	0/0/0	0/0/0
E2	0/0/0	0/0/0
E3	0/0/0	58/7/33
E4	0/0/0	0/0/0
E5	0/0/0	21/0/6
E6	0/0/0	15/5/19
E7	0/0/0	0/0/0

The SNP genotype of the father is heterozygous, and both the paternal grandmother and mother are homozygous and have the same SNP genotypes.

**Table 4:**
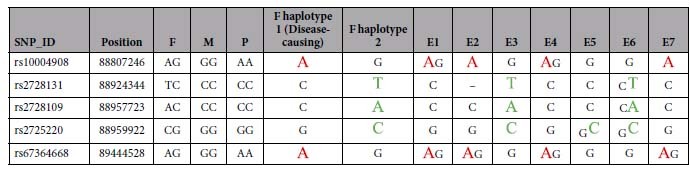
Sanger DNA sequencing results from the five SNP informative loci.

The SNP loci colored in red and green represent the informative SNPs that can determine a unique haplotype. The informative SNPs colored in red indicate the SNPs from haplotype 1 of the father. The informative SNPs colored in green indicate the SNPs from haplotype 2 of the father.

**Table 5:** Direct mutation detection results for the *PKD2* gene.

Sample	Targeted NGS	Sanger sequencing
P	–	heterozygous del
F	–	heterozygous del
E1	–	heterozygous del
E2	–	normal
E3	–	normal
E4	–	heterozygous del
E5	–	normal
E6	–	normal
E7	–	normal
