# Supplementary material for: Identification of PKD2 mutations in human preimplantation embryos in vitro using a combination of targeted next-generation sequencing and targeted haplotyping
Source: Sci Rep. 2016 May 6;6:25488. doi: 10.1038/srep25488 (PMC4858692; doi:10.1038/srep25488)
Supplement: Supplementary Information [file srep25488-s1.pdf]

Identification of *PKD2* mutations in human preimplantation embryos *in vitro* using a combination of targeted next-generation sequencing and targeted haplotyping

Song-Chang Chen[1, 2†], Xiao-Li Xu[3, 4†], Jun-Yu Zhang[1, 2], Guo-Lian Ding[1, 2], Li Jin[1, 2], Bei Liu[5], Dong-Mei Sun[3, 4], Chang-Lin Mei[6], Xiao-Nan Yang[3,4], He-Feng Huang[1, 2, 5], Chen-Ming Xu [1, 2, 5, *]

1. Institute of Embryo-Fetal Original Adult Disease Affiliated to Shanghai Jiao Tong University School of Medicine, 910 Hengshan Road, Shanghai 200030, P.R. China
2. International Peace Maternity and Child Health Hospital, Shanghai Jiao Tong University School of Medicine, 910 Hengshan Road, Shanghai 200030, P.R. China
3. BGI-Shenzhen, Shenzhen, 518083, P.R. China
4. Hangzhou Genomics Institute, 478 Wensan Road, Hangzhou 310012, P.R. China
5. Key Laboratory of Reproductive Genetics (Zhejiang University), Ministry of Education, 1 Xueshi Road, Hangzhou 310006, P.R. China
6. Division of Nephrology, Kidney Institute of CPLA, Changzheng Hospital, Second Military Medical University, Shanghai,200003, P.R.China

[†] These authors contributed equally to this work.

* Correspondence to: Dr. Chen-Ming Xu, Department of Reproductive Genetics, International Peace Maternity and Child Health Hospital, Shanghai Jiao Tong University School of Medicine, 910 Hengshan Road, Shanghai 200030, P.R. China; Phone: +86 21 64070434-30321; Fax: +86 21 64078219; E-mail: chenming_xu2006@163.com

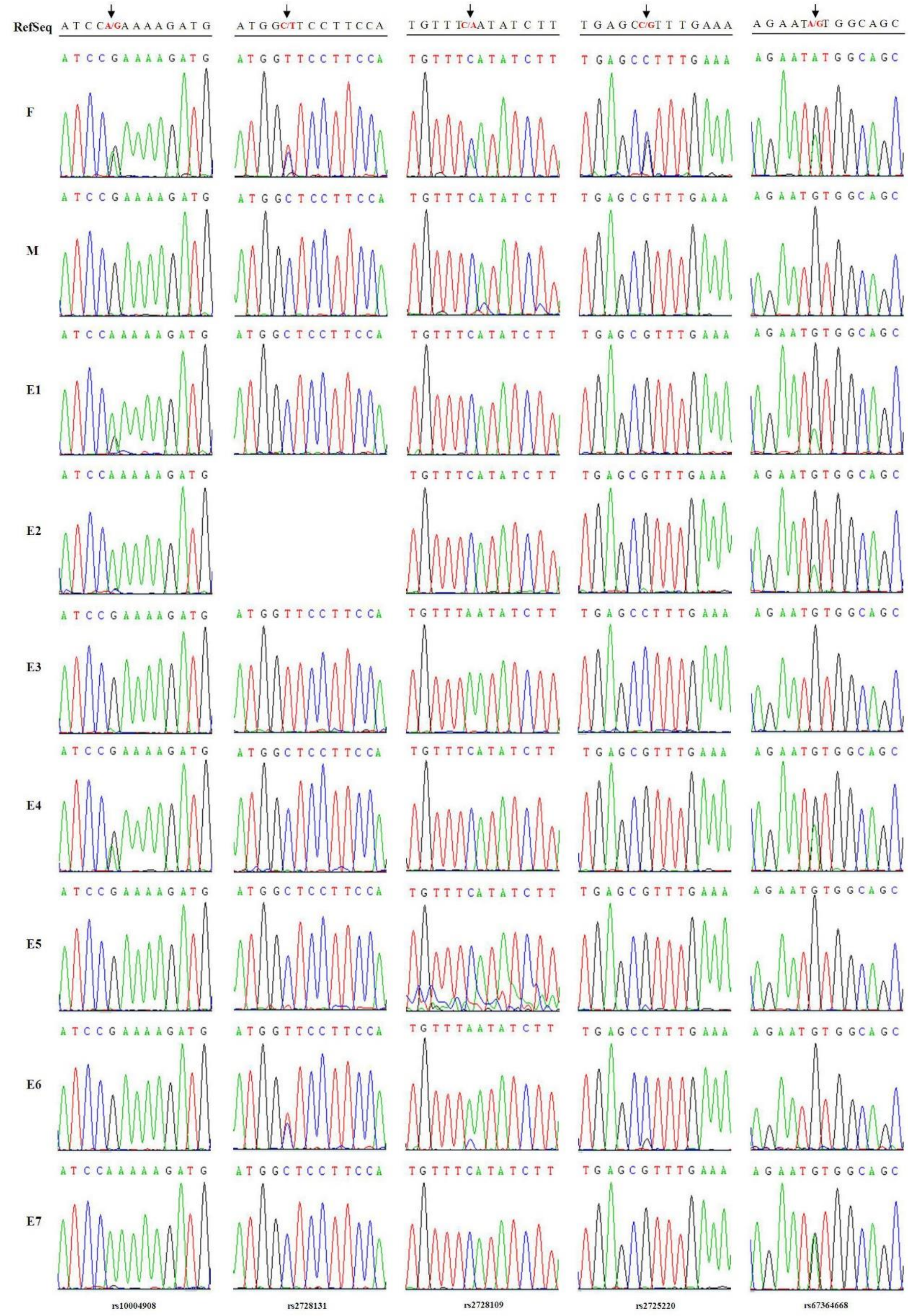

Supplementary information Figure S1. Direct detection results in five candidate SNPs by Sanger sequencing.

“F” represents “father”, “M” represents “mother” and “E” represents “embryo”. The arrows indicate the SNP locus. Tests were performed with both peripheral blood and single lymphocytes of the peripheral blood from the father, mother and a single cell obtained from a 6- to 8-cell cleavage-stage embryo on day three after IVF. We did not obtain the results of direct detection in rs2728131 of embryo 2 because of the invalid sequencing peak pattern obtained from Sanger sequencing.

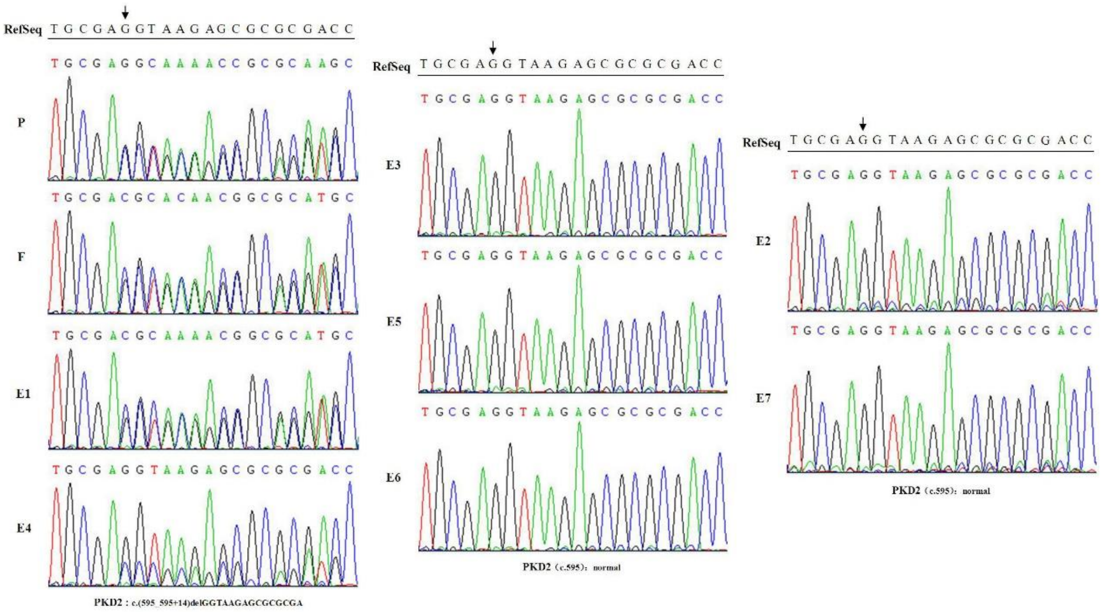

Supplementary information Figure S2. Paternal grandmother and embryos by Sanger sequencing.

"P" represents "paternal grandmother", "F" represents "father" and "E" represents "embryo". The arrows indicate the loci of the *PKD2* gene (c.595). There were heterozygous deletions in the *PKD2* gene c.595_595+14delGGTAAGAGCGCGCGA in the paternal grandmother, father, embryo 1 and embryo 4. The direct mutation detection results in the *PKD2* gene (c.595) of embryos 3, 5 and 6 were normal and were consistent with the results of family-specific haplotyping and embryo analysis. The direct mutation detection results in the *PKD2* gene (c.595) of embryos 2 and 6 were also normal but were inconsistent with the results of family-specific haplotyping and embryo analysis.

Table S1. Sex identification results using NGS.

	F	M	P	E1	E2	E3	E4	E5	E6	E7
Mean depth of chr X	14.89	37.25	35.85	22.43	38.69	26.83	31.35	41.36	0.12	37.13
Mean depth of chr Y	16.47	0.21	0.18	13.91	0.05	10.76	0.06	0.01	18.69	12.98
Gender test result	M	F	F	M	F	M	F	F	?	M

Although embryo 6 was considered to be normal with PGD, the NGS data for the sex chromosomes indicated abnormality, which is colored in red. The results illustrated the great risk of having a missing X chromosome, and this finding was confirmed by karyotype analysis (data not shown).

An array-based gene chip was designed to capture all of the exons of 21 genes, approximately 2,000 SNP (Minor Allele Frequency, MAF > 0.3) loci regions around each gene and in the X and Y chromosome-specific regions. In addition to the *PKD2* gene, we used this method to detect other genes within the chip. The following tables show the analyses for two pedigrees:

Pedigree #1: The target gene was *DMD* (chr X). Three samples, including the husband (DMD normal), wife (*DMD* c.2280delA carried), daughter (*DMD* c.2280delA carried) and five embryos, were tested.

The informative SNPs were substantially and uniformly distributed around the *DMD* gene in all five embryos. See Supplementary information (Table S2). The haplotype analysis of the *DMD* gene in the five embryos was consistent with the results of the multiple fluorescence PCR (MF-PCR) method.

Table S2. Targeted NGS and haplotype analysis results in the embryos of pedigree #1 with the *DMD* gene.

Embryo	Gene	M haplotype 1	M haplotype 2	Haplotype identification	Sex identification	Detection results
E1	*DMD*	14/36/24	0	M1	female	carried
E2	*DMD*	0	10/22/8	M2	male	normal
E3	*DMD*	0	13/23/9	M2	male	normal
E4	*DMD*	0	10/23/12	M2	male	normal
E5	*DMD*	11/33/21	0	M1	male	abnormal

The proband (daughter) carried disease-causing mutations in the DMD gene. “M1” refers to the mother’s haplotype 1, which represents the mutation haplotype.

Pedigree #2: The target gene was *PKD1* (chr16). Three samples, including the husband (proband), the proband's mother, his wife (normal) and four embryos, were tested. Both the proband and his mother carried a heterozygous deletion in P.GLu313Ter(GAG>TAG) of the *PKD1* gene, but the proband's wife was normal.

Two out of the four embryos had severe amplification bias via WGA, and the overall genomic coverage was low (Table S3). The results of haplotype analysis (Table S4) were consistent with the results of the single-gene genetic disease detection.

Table S3. Single-gene genetic disease detection of pedigree #2 with the *PKD1* gene in embryos.

Embryo	10× Coverage of target region (%)	Qualification (yes/no)	Detection results
e1	45.12	No*	-
e2	70.69	Yes	Abnormal
e3	34.03	No*	-
e4	66.56	Yes	Normal

"*" represents embryos with severe amplification bias via WGA. The overall genomic coverage was low, and the *PKD1* gene could not be analyzed.

Table S4. Targeted NGS and haplotype analysis results of pedigree #2 with the *PKD1* gene in embryos.

Embryo	Gene	F1		F2 (P.GLu313Ter)		haplotype identification	Detection results
		upstream	downstream	upstream	downstream		
e2	*PKD1*	0	0	27	6	F2	abnormal
e4	*PKD1*	68	27	0	0	F1	normal

"F1" represents a normal haplotype;"F2" represents a mutation haplotype

Table S5. Sanger DNA sequencing results in five SNP informative loci from peripheral blood and a single lymphocyte from peripheral blood from the father.

SNP_ID	Position	Peripheral blood	Single lymphocyte from peripheral blood									Rate of ADO (%)	Average ADO (%)
			1	2	3	4	5	6	7	8	9		
rs10004908	88807246	AG	A	A	–	A	A	A	AG	AG	G	77.78	
rs2728131	88924344	TC	C	T	C	CT	CT	CT	CT	C	C	55.56	
rs2728109	88957723	AC	CA	A	A	C	CA	C	C	CA	C	66.67	57.78
rs2725220	88959922	CG	GC	C	GC	G	GC	G	GC	GC	GC	33.33	
rs67364668	89444528	AG	A	G	G	G	A	AG	AG	AG	AG	55.56	
